# Neuroligin 3 R451C mutation alters electroencephalography spectral activity in an animal model of autism spectrum disorders

**DOI:** 10.1186/s13041-017-0290-2

**Published:** 2017-04-07

**Authors:** Jackie J. Liu, Kevin P. Grace, Richard L. Horner, Miguel A. Cortez, Yiwen Shao, Zhengping Jia

**Affiliations:** 1 Neurosciences & Mental Health Program, The Hospital for Sick Children, 555 University Ave., Toronto, M5G 1X8 ON Canada; 2 Department of Physiology, Faculty of Medicine, University of Toronto, Toronto, Canada; 3 Department of Medicine, Faculty of Medicine, University of Toronto, Toronto, Canada; 4 Department of Paediatrics, Faculty of Medicine, University of Toronto, Toronto, Canada; 5 Division of Neurology, The Hospital of Sick Children, Toronto, Canada

**Keywords:** Nlgn3 R451C mouse model, Autism, EEG, NREM, REM, Sleep deficit

## Abstract

Human studies demonstrate that sleep impairment is a concurrent comorbidity of autism spectrum disorders (ASD), but its etiology remains largely uncertain. One of the prominent theories of ASD suggests that an imbalance in synaptic excitation/inhibition may contribute to various aspects of ASD, including sleep impairments. Following the identification of Nlgn3^R451C^ mutation in patients with ASD, its effects on synaptic transmission and social behaviours have been examined extensively in the mouse model. However, the contributory role of this mutation to sleep impairments in ASD remains unknown. In this study, we showed that Nlgn3^R451C^ knock-in mice, an established genetic model for ASD, exhibited normal duration and distribution of sleep/wake states but significantly altered electroencephalography (EEG) power spectral profiles for wake and sleep.

## Introduction

Autism spectrum disorders (ASD) are a class of pervasive neurodevelopmental conditions, affecting 1 in 110 children [1, 2]. ASD are diagnosed based on impaired social interaction, impaired communication, and restricted behavioural stereotypies. In addition, sleep impairment is reported as one of the most concurrent symptoms in this population [3, 4]; 40–80% of children with ASD are reported to have some sleep abnormalities [3–11]. Currently, sleep abnormalities in patients with ASD are largely undertreated as other symptoms take precedence [3]. However, studies show that core symptoms of ASD as well as behavioural measures are worsened by poor sleep quality [3], highlighting the importance to understand and treat sleep abnormalities in ASD. For example, the severity of social and communication deficits are exacerbated following sleep deprivation [3, 12, 13]. In addition, sleep impairments were also linked to increased aggression, irritability, hyperactivity, and affective problems, that may further impair daytime functioning of patients with ASD [3, 13–16]. Despite the pervasiveness of sleep impairment in the ASD population, its neurochemical underpinnings remain unclear.

ASD are usually diagnosed between the ages of 2 to 4, a developmental period with extensive activity-dependent neuronal remodelling, but their symptoms persist throughout adulthood [2, 17]. A prominent neurochemical notion for ASD etiology is the excitation/inhibition (E/I) imbalance theory which suggests that altered neuronal network excitability may underline ASD [2, 18, 19]. For example, while an upregulation of α-amino-3-hydroxy-5-methyl-4-isoxazolepropionic acid (AMPA) receptors, the principal mediator of fast excitatory synaptic transmission, was found in post-mortem brain samples of patients with ASD, a concomitant downregulation of the inhibitory γ-aminobutyric acid (GABA) receptors was found [2]. Furthermore, abnormal cortical and subcortical excitability was also proposed to be a critical contributor to sleep impairment in ASD [4].

Neuroligins (Nlgns), a family of postsynaptic cell adhesion proteins, are crucial for neuronal E/I balance through their regulation of GABAergic and glutamatergic synaptic strength [18, 20–23]. To date, 5 Nlgn family members have been identified in humans, including Nlgn 1-3, which have close homologs in mice [24, 25]. Importantly, a missense mutation in Nlgn3 (Nlgn3^R451C^), which results in its reduced synaptic expression due to increased retention in the endoplasmic reticulum [24, 26], is linked with ASD in humans [24, 25, 27] as well as impaired social interaction and vocal communication in rodent models [24, 28–30]. Although Nlgn3 is ubiquitously expressed in the brain, its function in synaptic transmission appears to be region-specific, affecting the somatosensory cortex and the hippocampus differently [24, 30]. Recently, a number of studies have looked at the role of neuroligins in sleep. In one study, Nlgn1 knockout mice were unable to maintain wakefulness and spent more time in NREM sleep [31] while drosophila deficient in neuroligin 4 exhibited impaired night sleep in another study [32]. To date, although numerous studies have demonstrated that Nlgn3^R451C^ mutation is linked to impaired social interaction and memory, whether it also contributes to sleep impairment is unknown. In this study, we employed electroencephalography (EEG)-electromyography (EMG) recordings to evaluate sleep properties in Nlgn3^R451C^ knock-in (KI) mice. We demonstrated altered EEG spectral profiles in wakefulness and sleep in these mice.

## Methods

### Animals, housing conditions, and genotyping

Adult Nlgn3^R451C^ mice obtained from the Jackson Laboratory were housed and bred under a 12 h light-12 h dark cycle (light on 6:40 am, light off 6:40 pm) with constant ambient temperature. Food and water were provided ad libitum. All animal protocols were approved by the Animal Lab Services ethics committee of the Hospital for Sick Children. To determine the genotype (wild type or knock-in) of the mice, the DNA fragments were amplified using polymerase chain reaction (PCR) with primers 5’- TGTACCAGGAATGGGAAGCAG-3’ and 5’- GGTCAGAGCTGTCATTGTCAC-3’ using the conditions recommended by the Jackson Laboratory.

### Stereotaxic surgery

Adult male mice [WT = 19.1 ± 1.0 (SEM) weeks, 33.4 ± 0.7 g, *n* = 7; Nlgn3^R451C^ KI = 19.3 ± 1.1 (SEM) weeks, 30.6 ± 0.6 g, *n* = 7] were used for surgery. Three cortical electrodes for EEG recordings and two insulated stainless steel wires (0.011 mm diameter, Cooner Wire, Chatsworth, CA, USA) for EMG recordings were soldered to a multi-channel connector (Digi-Key Electronics, Thief River Falls, MN, USA) prior to implantation. Animals were anesthetized using ketamine (100–150 mg/kg as needed, intraperitoneal, i.p.) and xylazine (7–10 mg/kg as needed, i.p.) prior to surgery. The three electrodes were inserted through holes made in the skull and placed against the dura, over the cerebellum, left frontal lobe (1.7 mm lateral to midline and 1.5 mm anterior to bregma), and right parietal lobe (1.7 mm lateral to midline and 1.0 anterior to lambda). In addition, 2 stainless steel wires were sutured into the neck muscles. Dental acrylic (Lang Dental Manufacturing Co., Inc., Wheeling, IL, USA) was used to secure the multi-channel connector to the skull. After surgery, ketoprofen (5 mg/kg, subcutaneous, s.c.) was administered for post-operative pain control and 1 ml 0.9% saline (s.c.) was administered for fluid loading.

### Experimental protocol and data acquisition

All mice were recovered for at least 8 days in their home cage and were habituated individually for another 3 days in the recording chamber prior to the 48 h EEG-EMG recording. Seven pairs of age-matched Nlgn3^R451C^ and their wildtype (WT) littermate were used for EEG-EMG recordings. The light-dark cycle during recording was kept consistent with the housing room condition. EEG and EMG signals were recorded, amplified, and filtered with HFF at 1 Hz and LFF at 90 Hz using Grasslab (Natus Neurology Incorporated – Grass Products, Warwick, RI, USA) and sampled at 512 Hz.

### Sleep wake states determination

**Fig. 1:**
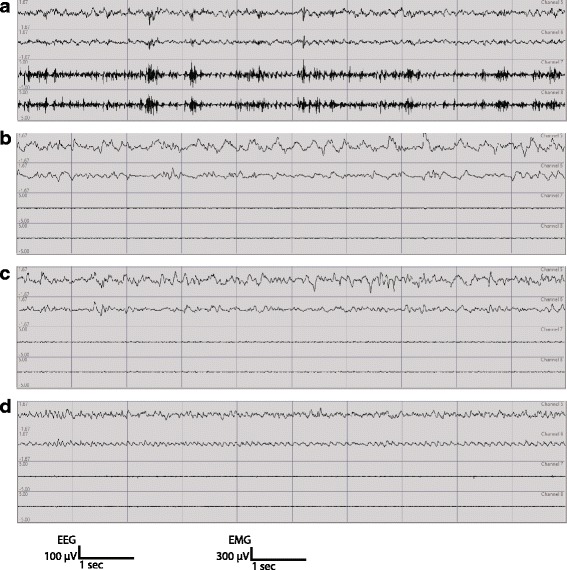
Representative traces of EEG-EMG recordings in a WT mouse. For each mouse, continuous recordings for 48 h were done (top 2 channels: EEG; bottom 2 channels: EMG). The top EEG channel is from left frontal electrode recording with reference to cerebellum. The second EEG channel is from right parietal electrode recording with reference to cerebellum. **a** Wake EEG is predominated by oscillations greater or equal to theta oscillations (≥5 Hz) with varying amplitude and is irregular in comparison to NREM and REM sleep. **b**, **c** NREM sleep is predominated by either high amplitude delta waves (<4 Hz) or mixed high amplitude theta/delta waves in the EEG channels and low muscle activity. The mixed high amplitude theta/delta waves in our mice often occur before transitioning into REM sleep. **d** REM sleep is characterized by regular low amplitude theta waves (≥7 Hz) in the EEG channels associated with muscle atonia. Every gridline marks 1 s in duration. The amplitude scale for EEG is 100 μV and the amplitude scale for EMG is 300 μV

The 48 h recordings were visually scored for sleep-wake states by independent investigators who were blind to the genotype of the mice. Sleep-wake states were identified by inspecting both EEG and EMG signals using 4-s epochs, and classified into wakefulness, NREM sleep, and REM sleep, as previously described [33]. Sample EEG-EMG traces of each sleep-wake state are shown in Fig. 1.

### EEG spectral analysis

The two EEG channels were subtracted to obtain a single EEG signal for Fast Fourier Transformation (FFT) using MatLab (MathWorks, Natick, MA, USA). These signals were further filtered to reduce noise prior to FFT and only frequency bands below 56 Hz were subjected to FFT. To achieve these, the filters were set as HFF = 0.55 Hz, band stop = 0.45 Hz, band stop = 60 Hz. Total EEG power (μV^2^) between 1 and 56 Hz for each 4 s epoch were obtained using MATLAB. To normalize the data, EEG power (μV^2^) for each frequency bin within each epoch was calculated as a percentage of the average of the total EEG powers (1–56 Hz) of all epochs.

### Statistical analysis

The EEG-EMG signals of each mouse were recorded for 48 h. The data from the 2 days were averaged. As noise in our recordings was characterized by very high amplitude oscillations, we removed all epochs with any frequency band that has a power of greater than 300% after normalization (regardless of frequency bands and sleep state, but most of these occur during wake states). The data were then re-normalized after the removal of these epochs. Once again, epochs with any 1 Hz frequency bin that has a normalized power > 300% were removed. This accounted for 2.1% of WT data and 2.2% of KI data. This was done to reduce the effect of extremely high amplitudes outlier oscillations on the overall data by reducing skewing effect. All the averaged data were stated as mean ± SEM and statistically evaluated by Student’s *t*-test for comparisons of two groups, or ANOVA (one-way, two-way or repeated measures wherever appropriate) for comparisons of more than two groups followed by post-hoc Bonferroni t-tests.

## Results

### Nlgn3^R451C^ mutant mice exhibit normal time-of-day distribution of sleep-wake states across time-of-day and sleep fragmentation

**Fig. 2:**
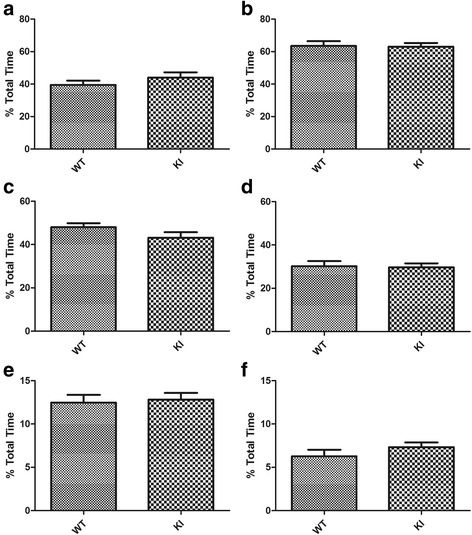
Total sleep-wake time in Nlgn3^R451C^ mutant mice. Proportion of total time spent in each vigilance state during the 12-h light and 12-h dark periods in WT (*n* = 7) and Nlgn3^R451C^ mice (KI, *n* = 7). Student *t*-test was used for this set of analysis. Both WT and Nlgn3^R451C^ mice spent more time in NREM and REM sleep during the light period than they did during the dark period. Nlgn3^R451C^ mice did not significantly alter the time that the mice spent in wake (**a**), NREM sleep (**c**), and REM sleep (**e**) during light period (all *p* >0.15. Nlgn3^R451C^ mice did not significantly alter the time that the mice spent in wake (**b**), NREM sleep (**d**), and REM (**f**) sleep during dark period (all *p* >0.28)

**Fig. 3:**
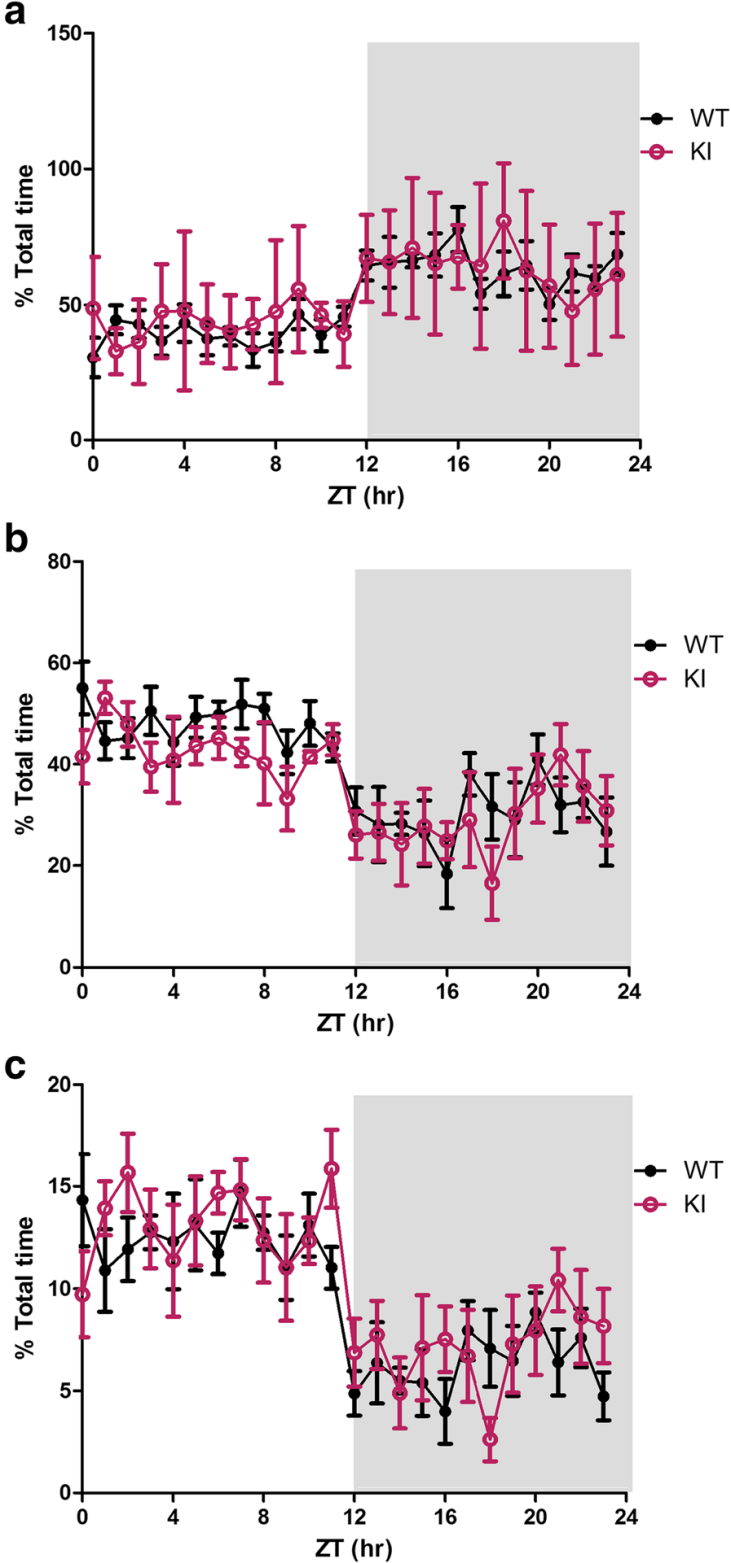
Time-of-day profile of vigilance states in WT and Nlgn3^R451C^ mutant mice. The distribution profile of each vigilance state across the entire recording. Nlgn3^R451C^ mutant mice exhibited a trend of less NREM sleep than WT (*p* = 0.051) (**b**), while the two groups did not differ from each other for wakefulness (**a**) and REM sleep (**c**) (genotype: both > 0.11)

**Fig. 4:**
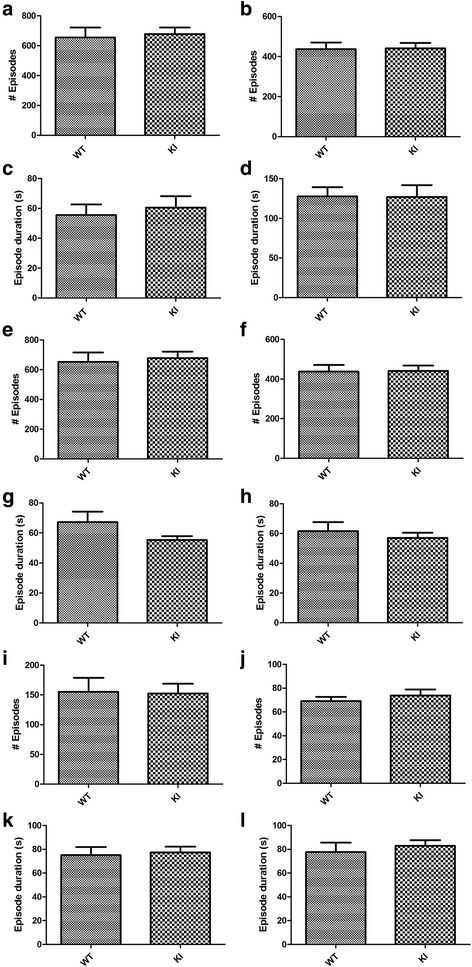
Sleep-wake episodes in Nlgn3^R451C^ mutant mice. Total number of episodes and the mean episode duration for each vigilance state were compared between WT (*n* = 7) and Nlgn3^R451C^ mice (KI, *n* = 7) using student *t*-test. Episode number of each state and their mean episode duration were separately assessed in the light (**a**, **c**, **e**, **g**, **i**, and **k**) or dark phase (**b**, **d**, **f**, **h**, **j**, and **l**). Nlgn3^R451C^ mutation did not result in significant alteration in the number of episodes (all *p* >0.45) nor the mean episode duration (all *p* >0.13) of wake (**a** to **d**), NREM (**e** to **h**), and REM sleep (**i** to **l**) in the light period or the dark period

We first analyzed if the proportion of time spent in each state differed between the first day and second day of recordings. As day 1 and day 2 did not differ significantly for any of the state (all *p* >0.098), the two days’ data were averaged for subsequent analysis. As patients with ASD often have shortened sleep duration, longer waking duration during the night, and altered circadian rhythm for sleep [3, 10, 14, 34], we analyzed the proportion of time that the mice spent in each vigilance state and their distribution during the light (ZT1-12 h) and dark (ZT13-24 h) periods. During both the light and dark periods, the Nlgn3^R451C^ mutant mice did not differ significantly from their WT littermates in the proportion of time that they spent in wakefulness, NREM sleep, and REM sleep (all *p* >0.14) (Fig. 2). To determine the effect of Nlgn3^R451C^ mutation on the distribution of vigilance states across the time-of-day, we analyzed the proportion of time spent during each hour over the 24 h recording period, and found no significant difference between genotypes for all three states (genotype: all *p* >0.05) (Fig. 3). As patients with ASD are often reported to have more fragmented sleep or more frequent awakenings after sleep onset than non-ASD subjects [3, 34], we also examined if Nlgn3^R451C^ mutant mice had alterations in the number of episodes and the duration of each individual sleep-wake episode. As shown in Fig. 4, there were no significant differences between Nlgn3^R451C^ mutant mice and WT controls in the number (all *p* >0.45) or the duration of sleep/wake episodes (all *p* >0.13) (Fig. 4). Taken together, these results suggest that Nlgn3^R451C^ mutation does not significantly affect the overall sleep/wake duration or contribute to fragmentation of sleep.

### Nlgn3^R451C^ mutant mice exhibit altered power spectral profiles for wakefulness, NREM, and REM sleep

**Fig. 5:**
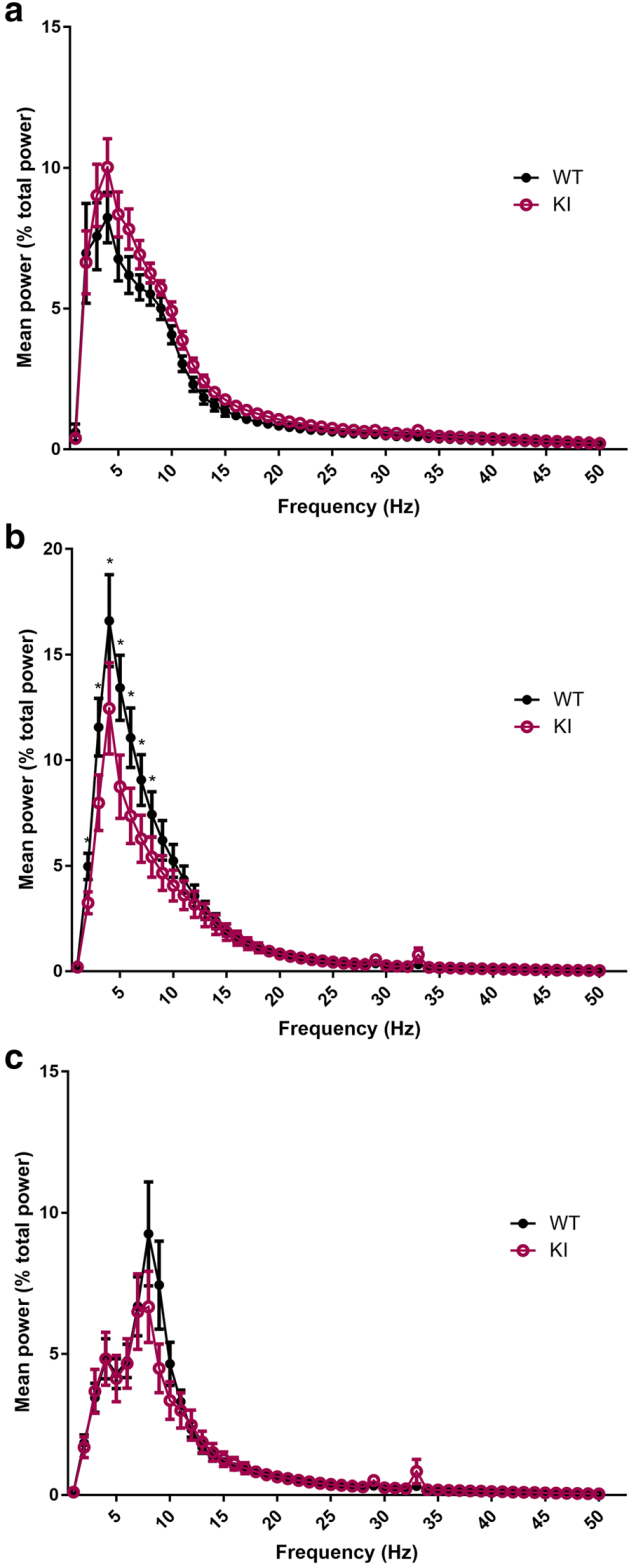
Power spectral profiles of WT and Nlgn3^R451C^ mutant mice. The power of the individual frequency band (1 Hz bins) was normalized by expressing it as % of the average of total power (1-56 Hz for all epochs). Repeated measure two-way ANOVA using “genotype” and “frequency” as factors revealed significant genotype-frequency interaction for NREM sleep (*F* = 2.856, *p* <0.001). The NREM (B) spectral profile of Nlgn3^R451C^ (KI) mice differed significantly between 2-8 Hz from WT mice (WT, *n* = 7; KI, *n* = 7; all *p* <0.05). More specifically, Nlgn3^R451C^ mice had reduced powers between 2-8 Hz during NREM sleep (**b**) in comparison to WT mice. No significant genotype effect or genotype-frequency interactions were found for wake (**a**) and REM (**c**) states

**Fig. 6:**
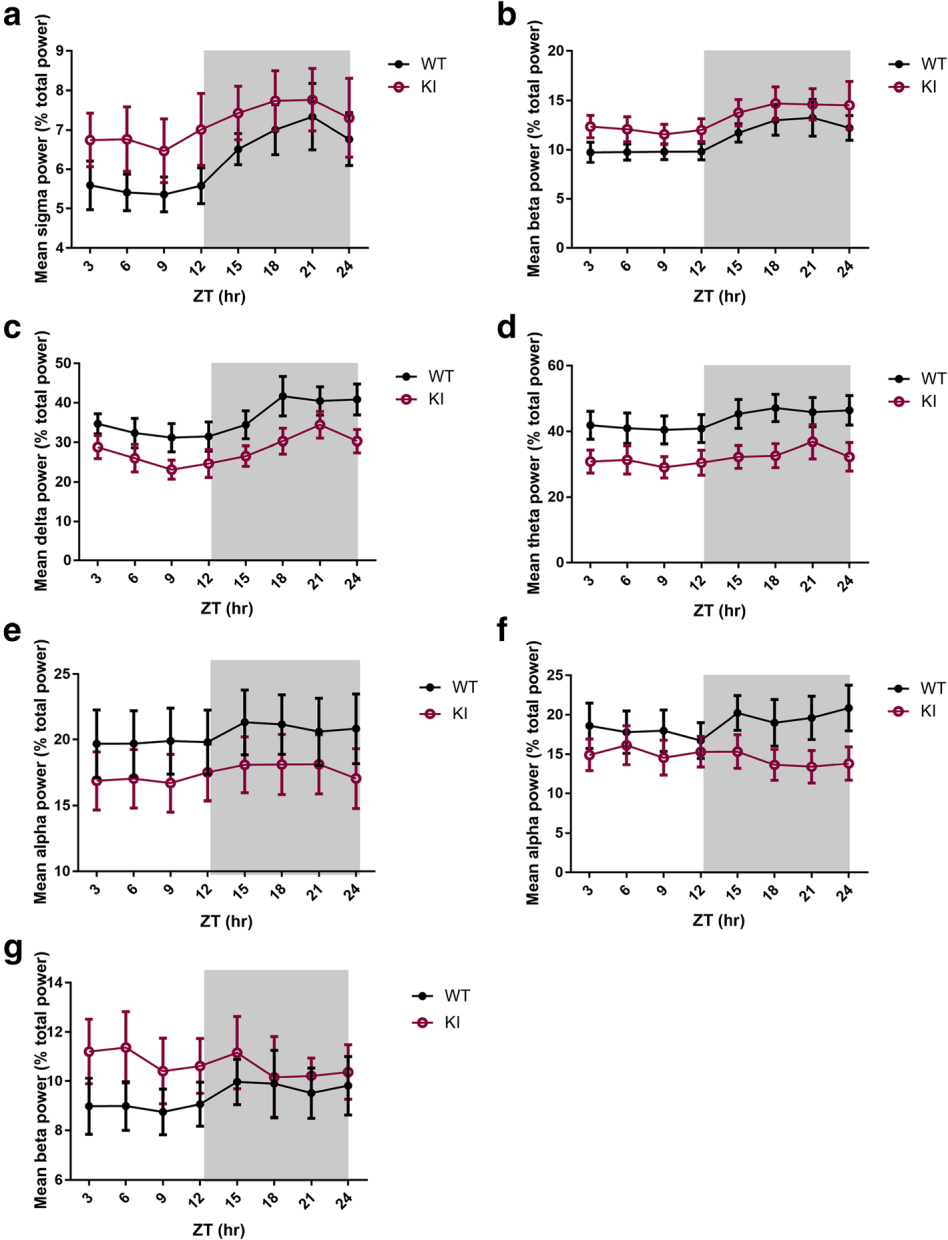
Altered power spectral profiles in Nlgn3^R451C^ mutant mice. The power of the individual frequency band was normalized by expressing it as % of the mean of total power (1-56 Hz for all epochs). The mean normalized power of each 3-h interval (of both days) is displayed over the 24-h period. “Genotype” and “time” were used as factors to examine their effects on delta, theta, alpha, sigma, and beta powers in each behavioural vigilance state (only data with significant genotype effect are displayed here). During wakefulness, Nlgn3^R451C^ (KI) mice exhibited significantly higher sigma (**a**) and beta (**b**) powers than WT mice (*p* <0.05). During NREM sleep, Nlgn3^R451C^ mice showed suppressed delta (**c**), theta (**d**), and alpha (**e**) powers than WT mice (*p* <0.05). During REM sleep, Nlgn3^R451C^ mice exhibited significantly lower alpha (**f**) and higher beta (**g**) powers than WT (*p* <0.05). No significant interactions between genotype and time were found (all *p* >0.05)

In order to examine the role of Nlgn3 in sleep quality, power spectrum of each state was compared between genotypes. Firstly, the mean spectral powers of 1 Hz bands in each vigilance state were normalized (to the average of the total power of all 1 Hz bins from 1 to 56 Hz across all epochs) and expressed as percentage of the total power. Significant genotype-frequency interaction was found for NREM state (*F* = 2.856, *p* <0.001) (Fig. 5). More specifically, post-hoc Bonferroni test showed significantly reduced EEG power between 2-8 Hz in Nlgn3^R451C^ mice (*p* <0.05) (Fig. 5). On the other hand, no significant “genotype” or “genotype-frequency” interactions were found for wake or REM sleep (both *p* >0.11) (Fig. 5). In addition, the normalized powers of delta (1–4 Hz), theta (5–8 Hz), alpha (9–12 Hz), sigma (13–15 Hz), and beta (15–30 Hz) frequency bands were examined in each sleep/wake state over time as these are indicators of cortical arousal and sleep quality [35–44]. These bands were normalized to the total power of 1–56 Hz as described above. These analyses revealed significantly altered wake/sleep EEG spectral profile in Nlgn3^R451C^ mutant mice. Specifically, the EEG sigma (*F* = 7.363, *p* <0.01) and beta (*F* = 9.024, *p* <0.01) powers were all significantly higher in Nlgn3^R451C^ mutant mice during wakefulness in comparison to WT mice (Table. 1, Fig. 6). Delta power during NREM sleep, which is an indicator of sleep depth [36], was reduced in Nlgn3^R451C^ mutants (*F* = 20.981, *p* <0.001) (Table 1, Fig. 6). Theta (*F* = 30.622, *p* <0.001) and alpha (*F* = 6.162, *p* <0.05) powers during NREM sleep were also reduced in Nlgn3^R451C^ mice (Table 1, Fig. 6). During REM sleep, while alpha power (*F* = 12.069, *p* <0.001) was reduced in Nlgn3^R451C^ mice, beta power was increased in Nlgn3^R451C^ mice (*F* = 4.829, *p* < 0.05) (Table 1, Fig. 6). During wakefulness, time had a significant main effect on beta power (*F* = 2.226, *p* <0.05) (Table 1). During NREM sleep, time had a significant main effect on delta power (*F* = 2.612, *p* < 0.05) (Table 1). Frequency bands with no main genotype effect (*p* >0.05) are not included in Fig. 6. In another set of analysis for time effect, we normalized the data of each frequency band by expressing it as a % of mean 24 h activity of each mouse for the targeted band within each arousal state. These analysis revealed significant time effect for all frequency bands (i.e. delta, theta, alpha, sigma, and beta; all *p* <0.001) in both genotypes. More specifically, EEG power for all of the stated bands were higher during the dark period for wakefulness, whereas EEG power for all of these bands were higher during the light period for NREM and REM sleep. Furthermore, we found no significant genotype-time interaction (all *p* >0.3), which is consistent with what we showed in Fig. 6 and in Table 1. This further supports that the effect of genotype on frequency bands is not time-dependent. The difference found on time effect between this analysis and the analysis done in Fig. 6 is likely due the fact that by normalizing the power of frequency band within each epoch to the total activity of the targeted band in the entire recording is more sensitive in revealing the time effect.

**Table 1:** Summary statistics for wake, NREM sleep, and REM sleep

	Df	Delta	Theta	Alpha	Sigma	Beta
Wake						
Gen	1	*F* = 2.317	*F* = 2.916	*F* = 1.224	*F* = 7.363	*F* = 9.024
		*p* = 0.131	*p* = 0.091	*p* = 0.271	*p* = 0.008**	*p* = 0.003**
Time	7	*F* =1.601	*F* =0.327	*F* =2.717	*F* = 1.597	*F* = 2.226
		*p* =0.144	*p* =0.940	*p* =0.013*	*p* = 0.146	*p* = 0.039*
Inter	7	*F* = 0.470	*F* = 0.092	*F* = 0.061	F = 0.136	*F* = 0.046
		*p* = 0.854	*p* =0.999	*p* = 1.000	*p* = 0.995	*p* = 1.000
NREM						
Gen	1	*F* = 20.981	*F* = 30.622	*F* = 6.162	*F* = 0.140	*F* = 0.457
		*p* <0.001***	*p* <0.001***	*p* = 0.015*	*p* = 0.709	*p* = 0.501
Time	7	*F* = 2.612	*F* = 0.623	*F* = 0.127	*F* = 0.221	*F* = 0.430
		*p* = 0.016*	*p* = 0.735	*p* = 0.996	*p* = 0.980	*p* = 0.881
Inter	7	*F* = 0.184	*F* = 0.120	*F* = 0.021	*F* = 0.023	F = 0.027
		*p* = 0.988	*p* = 0.997	*p* = 1.000	*p* = 1.000	*p* = 1.000
REM						
Gen	1	*F* = 0.885	*F* = 1.586	*F* = 12.069	*F* = 2.343	*F* = 4.829
		*p* = 0.349	*p* = 0.211	*p* < 0.001***	*p* = 0.129	*p* = 0.030*
Time	7	*F* = 0.350	*F* = 0.083	*F* = 0.120	*F* = 0.245	*F* = 0.115
		*p* = 0.928	*p* = 0.999	*p* = 0.997	*p* = 0.973	*p* = 0.997
Inter	7	*F* = 0.118	*F* = 0.134	*F* = 0.348	*F* = 0.401	*F* = 0.209
		*p* = 0.997	*p* = 0.995	*p* = 0.930	*p* = 0.899	*p* = 0.983

“Genotype” and “time” were used as factors to examine the effects of genotype (Gen) and time (3 h intervals) on spectral power for each behavioural state (WT *n* = 7, KI *n* = 7). The table displays the degree of freedom (Df), F values, and significance levels (*p*) for the factors and the genotype-time interactions (Inter) in the analysis. * *p* <0.05. ** *p* <0.01. *** *p* <0.001

## Discussion

In this study, we recorded EEG-EMG activity in the Nlgn3^R451C^ KI mice and their WT littermates to investigate whether the Nlgn3 ^R451C^ mutation is involved in sleep regulation. We found that both WT and Nlgn3^R451C^ mutant mice spent more time sleeping during the light phase compared to the dark, consistent with the typical time distribution of vigilance states in nocturnal rodents [40]. Nlgn3^R451C^ mutant mice were also not different from their WT controls in the total amount of time as well as the total number and durations of episodes for each sleep/wake state, suggesting that Nlgn3^R451C^ mutation alone may not be sufficient to cause reduced sleep time or frequent waking observed in patients with ASD [3, 4, 11]. However, Nlgn3^R451C^ mutant mice exhibited significantly altered EEG power spectra profiles, suggesting that this mutation may contribute to alterations in the quality of sleep/wake states.

During wakefulness, the Nlgn3^R451C^ mice exhibited increased sigma and beta spectral powers compared to WT. The significance of these changes is unknown. Delta power during NREM sleep was significantly reduced in Nlgn3^R451C^ mutant mice, which may reflect heightened arousal and ‘lighter’ sleep in the mutants [35, 45]. The mechanism for the reduced delta power is unknown, but it may be related to altered GABA inhibitory transmission. Delta oscillations are generated by the thalamo-corticol network and are regulated by GABAergic transmission [38, 46–49], and interestingly, a reduction in E/I ratio in the somatosensory cortex was reported in the Nlgn3^R451C^ mutant mice due to increased inhibitory synaptic transmission without changes in excitatory transmission in this region [24, 30]. Consistent with this finding, suppression of delta oscillations was also observed following the administration of diazepam, a GABA agonist [38, 50]. Theta oscillations, which are predominantly generated by the hippocampal network [40, 51, 52], were also reduced in Nlgn3^R451C^ mutant mice during NREM sleep. Previously, Nlgn3^R451C^ mutants mice were demonstrated to have increased excitatory glutamatergic synaptic transmission without alterations in inhibitory GABAergic transmission in the hippocampus, resulting in an augmented E/I ratio in this region [30]. Surprisingly, a reduction in E/I ratio via diazepam administration also reduced theta oscillations during NREM sleep [38]. As diazepam increases GABAergic transmission, which is not affected in the Nlgn3^R451C^ mutant hippocampus [30], these data suggest that Nlgn3 may regulate NREM sleep theta oscillations via mechanisms independent of GABAergic signalling. Alpha oscillations, which are believed to be generated by cortico-cortical and thalamo-cortical networks [40], were reduced during both NREM and REM sleep in Nlgn3^R451C^ mutant mice. Alpha oscillations are thought to reflect input from dorsal anterior cingulate cortex, anterior insula, and thalamus that relay sensory information to the cortex, signalling the brain of external stimuli [43, 53]. Increased alpha power during REM sleep therefore may reflect micro-arousal during REM sleep and possibly contributing to REM sleep instability [39, 43, 54]. Hence, the reduced alpha power in Nlgn3^R451C^ KI mice might indicate more stable REM sleep in these mutants. Although the significance of alpha power reduction for sleep/wake regulation remains unclear, it is consistent with the increased cortical GABAergic transmission as diazepam also suppresses alpha oscillations [50]. In human, beta oscillations (15-30 Hz) are reflective of cortical arousal within sleep [37, 45]. Although, the changes in beta power in patients with insomnia are variable during REM sleep [45], several reports have demonstrated increased beta power in REM sleep in patients with primary insomnia [37, 41, 55]. It is plausible the increased beta oscillations during REM sleep may also suggest poor sleep quality in Nlgn3^R451C^ mice. The simultaneously reduced alpha power and increased beta power may indicate the simultaneous activation of both wake-promoting and sleep-promoting mechanisms in Nlgn3^R451C^ mice.

It is interesting to note that the alterations in spectral powers in Nlgn3^R451C^ mice are quite different from those found in Nlgn1KO mice [31]. For example, Nlgn1KO mice showed decreased high delta, theta, alpha powers during wakefulness while showing a trend of increased delta power during NREM, whereas Nlgn3^R451C^ mutant mice exhibited increased sigma and beta bands during wakefulness and reduced delta, theta, and alpha powers during NREM sleep. These results suggest that the role of neuroligins in sleep/wake regulation is dependent on specific members of the neuroligin gene family. Consistent with this possibility, members of the neuroligin family show different patterns of expression and modes of regulation [21, 22]. However, it is possible that in Nlgn3^R451C^ mutant mice, there may be developmental compensations that alter the expression of other proteins, including Nlgn 1, which could complicate the interpretation of the results. It is also possible that members of the neuroligin family cross-talk so that the deletion of one member affects the functionality of the others. Further studies are needed to elucidate whether and how these interactions and specificity are achieved and their impact on sleep/wake states by using acute manipulations and a combination of knockout mice lacking one or more neuroligins. Lastly, we found a trend of reduced NREM sleep in Nlgn3^R451C^ mice (Fig. 3). Future studies using higher number of mice may help better elucidate the effect of Nlgn3^R451C^ on NREM sleep.

## Abbreviations

AMPA: α-amino-3-hydroxy-5-methyl-4-isoxazolepropionic acid
ASD: Autism spectrum disorders
E/I: Excitation to inhibition
EEG: Electroencephalography
EMG: Electromyography
GABA: γ-aminobutyric acid
i.p.: Intraperitoneal
KI: Knock-in
Nlgn: Neuroligin
NREM: Non-rapid-eye-movement
REM: Rapid-eye-movement
s.c.: subcutaneous
WT: Wild-type
